# Analysis of the clinical efficacy of tumor resection methods used in 20 patients with clavicular tumor

**DOI:** 10.1186/s12957-019-1642-4

**Published:** 2019-06-17

**Authors:** Yun Liu, Xian-Ying Huang, Wen-Yu Feng, Xiao-Ting Luo, Chang-Wu Wei, Jian-Hong Liu, Yin-Juan Lai, Zeng-Ming Xiao, Xiao Zhang, Xin-Li Zhan

**Affiliations:** 1 Department of Orthopaedics, First Affiliated Hospital of Guangxi Medical University, Nanning, Guangxi China; 2 Department of Neurology, First Affiliated Hospital of Guangxi Medical University, Nanning, Guangxi China; 3 Department of Pharmacy, First Affiliated Hospital of Guangxi Medical University, Nanning, Guangxi China; 4 Guangxi Medical University, Nanning, Guangxi China

**Keywords:** Tumor of clavicle, Claviculectomy, Reconstruction, Plate-cement complex

## Abstract

**Background:**

To retrospectively analyze the tumor resection method used in 20 patients with clavicular tumors and evaluate its clinical efficacy.

**Methods:**

A total of 9 patients with clavicular benign tumors underwent intracapsular resection, and 11 patients with clavicular malignant tumors underwent tumor resection from May 2012 to May 2017. Of the 11 patients, 5 underwent clavicular reconstruction using the plate-cement complex. Surgical efficacy was assessed using the Musculoskeletal Tumor Society, Constant-Murley, and American Shoulder and Elbow Surgeons shoulder outcome scores preoperatively until 12 months postoperatively.

**Results:**

The average duration of follow-up care was 33.7 (12–71) months. Of the 20 patients, 3 patients died, 3 survived with tumor recurrence or metastasis, and 14 survived with no tumor recurrence. Among the 5 patients who underwent resection of malignant clavicular tumors and reconstruction, 2 underwent a re-operation because of a loose screw and plate displacement. In the functional assessment of the shoulder joint, patients with benign and malignant clavicular tumors showed significantly higher scores postoperatively compared with preoperative scores. For malignant clavicular tumors, no significant improvement was observed when comparing the non-reconstruction and reconstruction groups.

**Conclusions:**

Surgery is an optimal treatment for clavicular tumors. In patients with benign clavicular tumors, simple intracapsular resection can achieve a satisfactory prognosis. Reconstruction of a clavicular defect after resection of a clavicular malignant tumor is not recommended.

## Background

The clavicular tumor is a rare bone tumor constituting different kinds of pathological patterns. Plasma cell tumor and osteochondroma are the most common types of clavicular malignant and benign tumors, respectively [1]. The main clinical symptoms include pain and local masses. Additionally, the presenting pain will be more notable and severe if the patient simultaneously suffers from pathological clavicular fractures. At present, a partial or total resection of the clavicle is considered the most optimal treatment option [2–6].

The clavicle is an S-shaped, slender, and subcutaneous bone. It is connected to the sternal shaft and the acromion, which play a part in constituting the sternoclavicular and acromioclavicular joints, respectively [7, 8]. Some studies revealed that the range of claviculectomy did not affect the function of the shoulder joint; thus, its action usually demonstrated a more favorable prognosis in a short- to medium-term follow-up postoperatively [3, 9]. However, a unified guideline on the surgical treatment protocol is lacking, and the number of clavicular tumor case is small because of its low incidence [3, 9–11]. For benign clavicular tumors, it is acknowledged that autologous bone grafting after intracapsular scraping or complete tumor resection can result in an ideal outcome [5, 6]. However, the postoperative effect assessment is rarely considered, especially on the functional evaluation of the shoulder joint. In the case of clavicular malignant tumors, a partial or total resection is considered the most appropriate treatment option. Despite the favorable options, whether or not clavicular defects should be reconstructed remains controversial. In order to restore the shape of the clavicle, various materials have been used to reconstruct the defects, such as allogeneic bone, autologous bone, and bone cement [2, 12–17]; however, fewer studies have been conducted on the usage of plate-cement complex or its efficacy.

This study aimed to retrospectively analyze patients who underwent resection of clavicular tumors and evaluate its clinical efficacy. In addition, we investigated the different outcomes between the clavicular reconstruction and non-reconstruction groups in malignant clavicular tumor patients.

## Methods

Our study included 20 patients (12 men and 8 women, aged 33.2 on average [range 5–56 years]) with clavicular tumors, who were recruited from the Department of Spine at the First Affiliated Hospital of Guangxi Medical University, China, from May 2012 to May 2017. Written informed consent was obtained from each patient, and this study was approved by the Ethics Committee of the First Affiliated Hospital of Guangxi Medical University.

Two senior physicians conducted tumor size measurements, using X-ray/CT, and surgical treatments for all patients. Whether or not a reconstruction of the shoulder was advisable for malignant tumor patients was determined based on the preoperative imaging examination and the patients’ obvious signs of shoulder joint instability. All patients were divided into malignant tumor group (including 3 plasmacytomas, 2 osteosarcomas, 2 Ewing sarcomas, 1 synovial sarcoma, and 3 metastatic cancers) and benign tumor group (including 4 osteochondromas, 2 simple bone cysts, 2 ossifying fibroids, and 1 spindle cell mesenchymal tumors) according to preoperative pathological examination results from the puncture biopsy. The malignant tumor group was divided further into reconstruction and non-reconstruction groups based on whether the clavicle was reconstructed. Moreover, different surgical procedures were performed according to each patient’s histopathological results: 9 patients with clavicular benign tumors underwent intracapsular resections, and 11 patients with malignant tumors underwent total tumor resections. In addition, 5 of the 11 patients with malignant tumors underwent clavicular reconstruction using the plate-cement complex (combination of titanium plates and bone cement). Finally, 1 patient received chemotherapy and 3 patients received radiotherapy postoperatively.

### Surgical methods

#### Surgical treatment of benign clavicular tumors

**Fig. 1:**
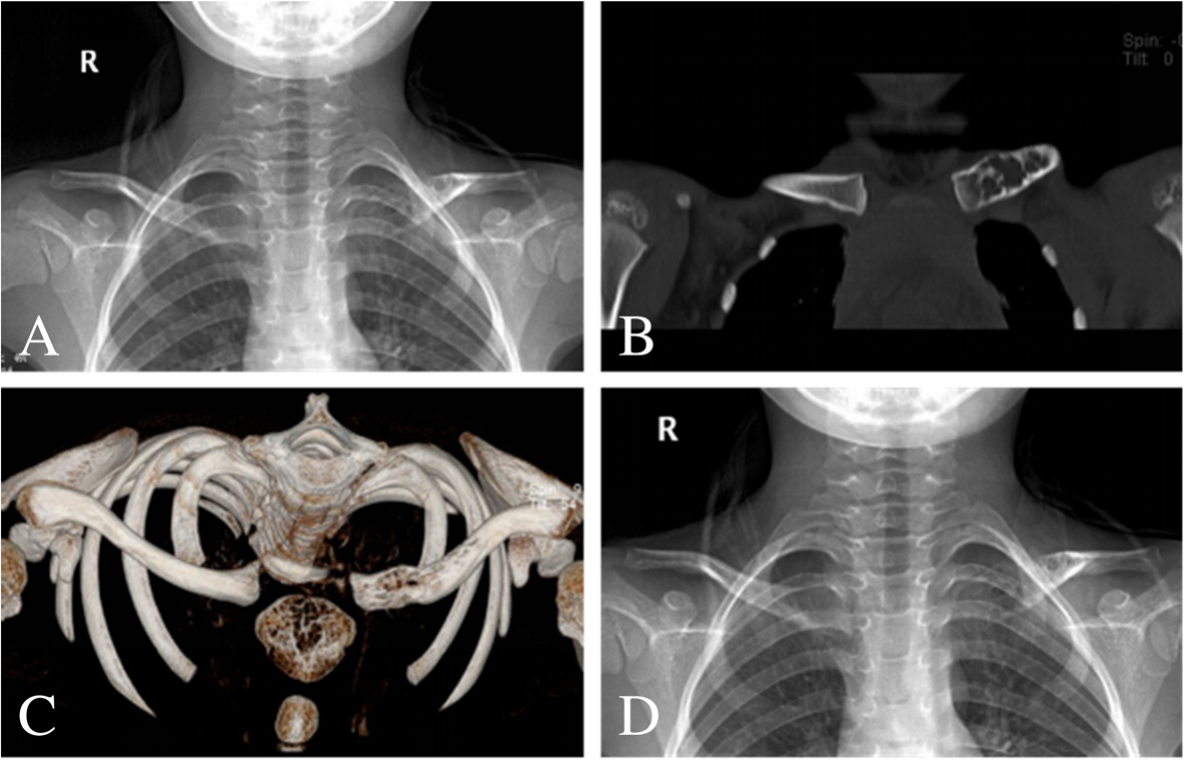
A 5-year-old male with left, medial, and clavicular simple bone cysts. **a** Preoperative plain X-ray. **b**, **c** CT shows an obvious tumor destruction on the medial left clavicle. **d** Postoperative X-ray

An arc incision was made on the surface of the clavicle, the skin and subcutaneous tissue were cut open, the clavicle was exposed at the tumor site, a small incision was made in the tumor wall with a bone knife, and the damaged collarbone tissue was fully scraped away with a spatula. After adequate hemostasis, the incision was closed and the removed tissue was sent for pathological examination (Fig. 1).

#### Surgical treatment of clavicular malignancy

**Fig. 2:**
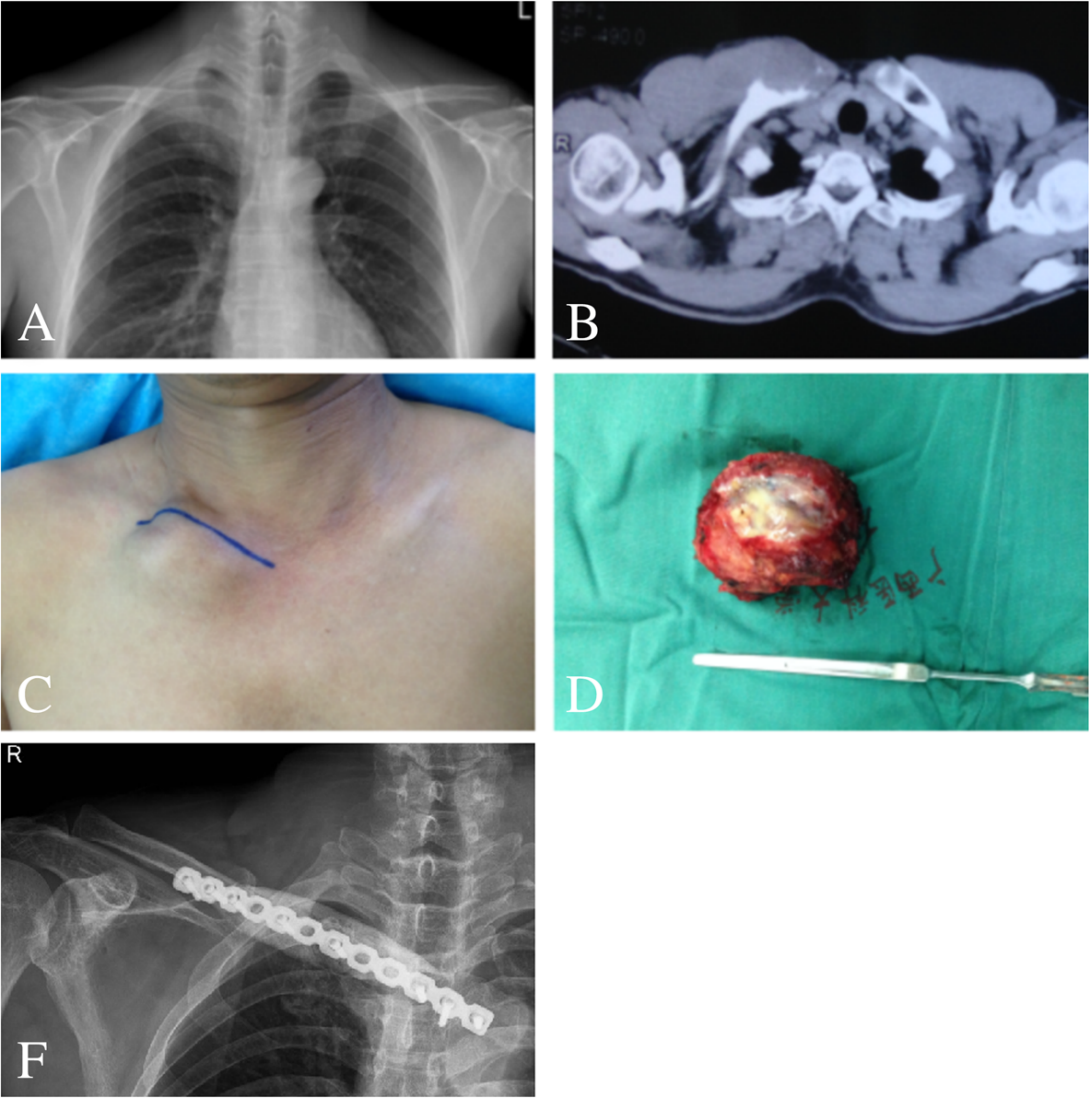
A 46-year-old man with right, medial, and clavicular synovial sarcoma. **a**, **b** Preoperative plain X-ray and CT show an obvious tumor destruction on the medial right clavicle. **c**, **d** General view of the tumor during surgery. **f** Postoperative X-ray shows the plate-cement complex is well fixed

**Fig. 3:**
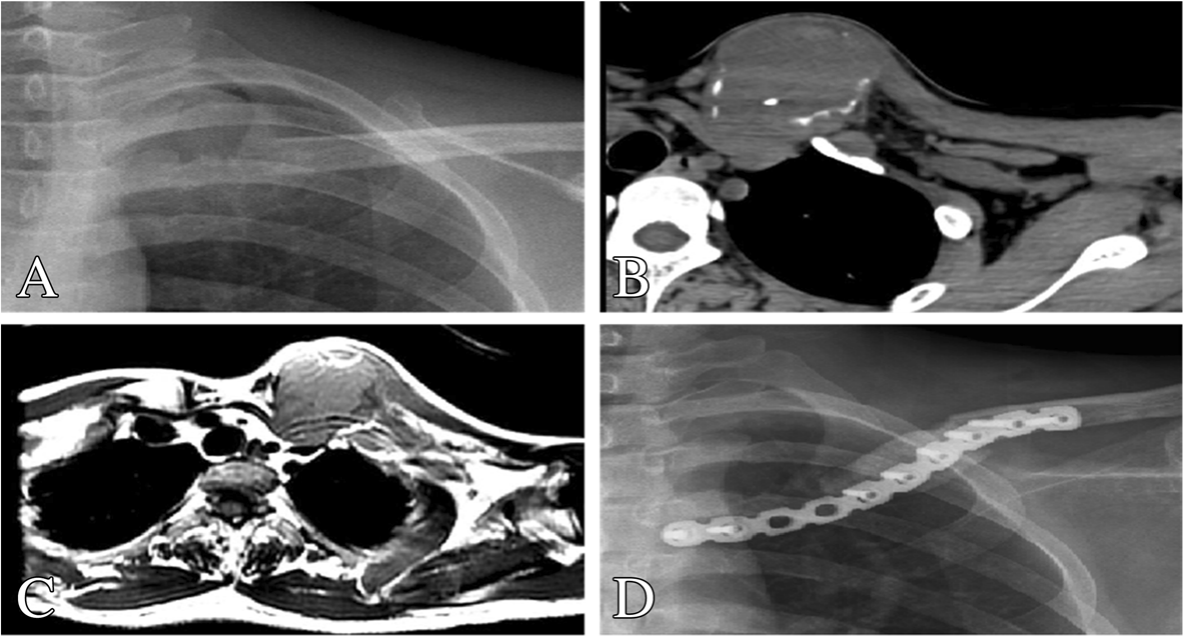
A 55-year-old man with left, lateral, and metastatic cancer. **b** Preoperative plain X-ray. **b**, **c** CT and MRI show an obvious tumor destruction on the medial left clavicle. **d** Postoperative X-ray 1 year after surgery

An arc incision was made on the surface of the clavicle, the skin and subcutaneous tissue were cut open, and the deep fascia was separated layer by layer. The tumor of the clavicle bone was obtuse; it was separated and exposed with a periosteal dissection device, and the surrounding blood vessels, tissues, and distal clavicle were explored for tumor invasion. The clavicle was cut off 2 cm from the edge of the tumor, or the sternoclavicular or acromioclavicular joints were cut off to completely remove the tumor (Fig. 2). For the non-reconstructive group, the incision was closed after adequate hemostasis and the removed tissue was sent for pathological examination. For the reconstruction group, after tumor resection, the shape of the clavicle was reconstructed using the plate-cement complex: an anatomic bone plate 3.5 mm arc reconstruction titanium plate (West Vein, China) was inserted, and 2–3 cortical bone screws were applied to fix it on the proximal and distal clavicle stump or gladiolus, respectively. Soft bone cement (J&J, USA) was applied to cover the soft clavicle defect, and 2–3 cortical screws were applied to fix the soft bone cement on the soft steel plate (Fig. 3).

### Follow-up and statistical analysis

#### Follow-up

Routine follow-up and re-examinations, using plain X-ray, CT, or MRI, were conducted postoperatively on all patients in the 1st, 3rd, 6th, and 12th months followed by once annually. The shoulder joint function was evaluated using the Musculoskeletal Tumor Society (MSTS) score [18], Constant-Murley score [19], and American Shoulder and Elbow Surgeons (ASES) shoulder outcome score [20] preoperatively and in the 12th month postoperatively.

#### Statistical analysis

All analyses were performed using the SPSS 22.0 software package. Differences in evaluating shoulder joint function preoperatively and postoperatively were assessed using the paired 2-sample *t* test. In patients with malignant tumors, the treatment effects between the reconstruction and non-reconstruction groups were compared using independent 2-sample *t* tests. *P* < 0.05 was considered statistically significant.

## Results

### The survival condition of patients with clavicular tumors

Twenty patients diagnosed with clavicular tumors were recruited in this study. The characteristics of the tumors, surgical procedures, final pathological diagnoses, therapeutic effects, and survival condition are presented in Table 1. The average duration of follow-up care was 33.7 (range 12–71) months. After 23 months postoperatively, 1 patient died from local tumor recurrence and pulmonary metastasis. Out of the 20 patients, 2 had primary lung cancer and died in the 15th and 23rd months postoperatively, respectively. One other patient had a local recurrence, and 2 presented with lung and skeletal metastases. The remaining 14 patients achieved a disease-free status.

**Table 1:** Summary of demographic and clavicular tumor characteristics for the patients

Patient	Gender/age (years)	Pathological diagnosis	Tumor location	Tumor size (cm)	Treatment	Follow-up (months)	Recurrence/metastasis	Result
Benign tumors								
1	F/8	Osteochondroma	Medial 1/3 (right)	2.5 × 2.5	Intracapsular resection	64	No/no	Survival
2	F/17	Osteochondroma	Lateral (left)	2.0 × 1.0	Intracapsular resection	35	No/no	Survival
3	F/29	Osteochondroma	Medial (left)	1.8 × 2.5	Intracapsular resection	12	No/no	Survival
4	M/9	Osteochondroma	Medial 1/3 (left)	2.0 × 3.0	Intracapsular resection	24	No/no	Survival
5	M/5	Simple bone cysts	Medial (left)	2.3 × 1.0	Intracapsular resection	71	No/no	Survival
6	F/12	Simple bone cysts	Medial 1/3 (right)	2.0 × 1.5	Intracapsular resection	57	No/no	Survival
7	M/15	Ossifying fibroids	Middle (right)	3.2 × 2.5	Intracapsular resection	30	No/no	Survival
8	M/10	Ossifying fibroids	Middle (right)	4.5 × 3.0	Intracapsular resection	57	No/no	Survival
9	M/34	Spindle cell mesenchymal tumor	Medial (left)	5.0 × 4.0	Intracapsular resection	57	No/no	Survival
Malignant tumors—non-construction group								
10	M/45	Plasmacytoma	Medial (right)	5.5 × 3.7	Tumor resection	23	No/no	Survival
11	M/43	Osteosarcoma	Medial 1/3 (right)	10.5 × 10.0	Tumor resection + radiotherapy	19	No/yes	Survival
12	M/53	Ewing sarcoma	Middle (right)	6.5 × 4.8	Tumor resection + radiotherapy	47	No/no	Survival
13	M/46	Metastatic cancer	Medial 1/3 (left)	7.0 × 6.8	Tumor resection + chemotherapy	23	No/no	Death
14	F/50	Metastatic cancer	Medial 1/3 (right)	7.5 × 6.0	Tumor resection	17	No/no	Survival
15	F/40	Ewing sarcoma	Lateral (left)	6.0 × 4.5	Tumor resection + radiotherapy	38	No/no	Survival
Malignant tumors—reconstruction group								
16	M/49	Plasmacytoma	Medial (left)	5.7 × 3.0	Tumor resection + plate-cement complex	23	Yes/no	Survival
17	F/42	Osteosarcoma	Medial (right)	5.5 × 5.0	Tumor resection + plate-cement complex	21	No/no	Survival
18	M/46	Synovial sarcoma	Medial (right)	5.5 × 4.5	Tumor resection + plate-cement complex	23	Yes/yes	Death
19	F/56	Plasmacytoma	Lateral (right)	4.8 × 1.9	Tumor resection + plate-cement complex + chemotherapy	18	No/yes	Survival
20	M/55	Metastatic cancer	Lateral (left)	4.5 × 2.5	Tumor resection + plate-cement complex	15	No/no	Death

*M* male, *F* female

### Benign clavicular tumors

Nine patients with benign clavicular tumors underwent intracapsular resection. Therefore, the structure of the clavicle was not damaged during the operation and its function remained normal. During the follow-up period, none of those patients had tumor recurrence (Table 1). Compared with the preoperative evaluations of shoulder function, patients showed significantly higher scores in the MSTS (59.63 ± 5.88 vs. 85.56 ± 4.71, *P* < 0.001), Constant-Murley score (49.67 ± 7.58 vs. 80.56 ± 5.10, *P* < 0.001), and ASES (46.11 ± 3.69 vs. 89.89 ± 3.69, *P* = 0.007) assessments 12 months postoperatively (Table 2).

**Table 2:** Comparison of the preoperative and postoperative functional assessment in the patients with clavicular benign tumors

Group/number	MSTS (%)	ASES	Constant-Murley score
Preoperation/9	59.63 ± 5.88	46.11 ± 3.69	49.67 ± 7.58
Postoperation/9	85.56 ± 4.71	89.89 ± 3.69	80.56 ± 5.10
*P*	< 0.001	0.007	< 0.001

*MSTS* Musculoskeletal Tumor Society score, *ASES* American Shoulder and Elbow Surgeons shoulder outcome score

### Clavicular malignancy

Wide excision of the mass with (patients 16–20) and without (patients 10–15) bone reconstruction was performed in patients with malignant clavicular tumors.

Patient 10, who was misdiagnosed with benign clavicular tumor and underwent intracapsular resection, suffered from local recurrence and had a re-operation 5 months later. He survived and presents no relapse or metastasis. Patient 11 presented with a large primary mass (measuring 10.5 × 10.0 cm) and pathological fracture and underwent complete mass resection and radiotherapy; however, pulmonary and C2, L2, and L5 vertebral metastases were found during the 19-month follow-up. Tumor excision and radiotherapy were performed on patients 12 and 15. They survived and remained free of local recurrence or metastases at the time of writing. The patients 13 and 14, whose primary tumors were lung carcinoma and breast cancer, respectively, underwent excision of clavicular tumors. Patient 13 also had chemotherapy postoperatively. None of them had local recurrence or metastasis.

Patient 16 presented with local recurrence 15 months postoperatively, but no signs of metastasis were observed. Neither recurrence nor metastasis was found in patient 17. Patients 18 and 19 were complicated with loose screws and displacement of the plate-cement complex after the reconstruction. Furthermore, patient 18 developed recurrence and metastasis. Patient 20, whose primary tumors were lung carcinomas, died without local recurrence after the 15-month follow-up.

In the functional evaluation of the shoulder joint, the postoperative MSTS, ASES, and Constant-Murley scores of all patients with clavicular malignant tumors were significantly higher than those preoperative, all *P* < 0.05 (Table 3), while there was no significant difference between the non-reconstruction and reconstruction groups, all *P* > 0.05 (Table 3).

**Table 3:** Comparison of the reconstruction and non-construction functional assessment in the patients with clavicular malignant tumors

Groups/number	MSTS			ASES			Constant-Murley score		
	Preoperative (%)	Postoperative (%)	*P*	Preoperative	Postoperative	*P*	Preoperative	Postoperative	*P*
Reconstruction/5	59.33 ± 3.65	86.00 ± 5.96	0.043	46.60 ± 7.83	91.60 ± 3.13	0.043	49.00 ± 6.16	81.00 ± 3.39	< 0.001
Non-reconstruction/6	58.89 ± 2.72	86.11 ± 4.91	< 0.001	43.00 ± 6.26	87.17 ± 5.27	< 0.001	45.17 ± 6.11	79.67 ± 3.08	< 0.001
*P*	0.822	0.974		0.418	0.134		0.331	0.511	

## Discussion

### Characteristics of clavicular tumors

Due to the significantly different developmental patterns of the clavicle compared with other tubular bones and its unique morphological structure, the clavicular tumor is a rare disease with a reported incidence of 0.45–1.01% of all bone tumors [21]. A total of 1756 patients diagnosed with bone tumors were treated in our hospital from May 2012 to May 2017. Among them, only 20 had clavicular tumors. Thus, the incidence rate was 1.14% in our hospital, which was slightly higher than the rates in other reports.

Benign clavicular tumors are more common in adolescents, while malignant tumors tend to develop in older people. In our study, 9 patients with benign tumors were < 35 years old, and the remaining 11 patients with malignant tumors were older than 40 years old (average age: benign vs. malignant = 15.44 ± 9.86 vs. 47.73 ± 5.33 years, *P* < 0.001). Our results were consistent with the previous literature reports [1, 10]. This led us to speculate that advanced age may be a risk factor for the occurrence of malignant clavicular tumors.

Most of the pathological types of bone tumors can occur in the clavicle. Plasma cell tumors and osteochondroma are the most common types of malignant and benign clavicular tumors, respectively [1]. Our pathological results were in agreement with the characteristics of clavicular tumors in East Asia [1].

### Surgical methods for benign clavicular tumor

The symptoms of clavicular tumors include local pain and swelling, which can seriously impair the stability and function of sternoclavicular and acromioclavicular joints and influence daily life. Surgery was regarded as a more optimal treatment option [2, 3, 11, 22, 23]. However, the standard for operative management remains controversial.

Benign clavicular tumors show a smaller volume, intact capsule, less invasion, and lower incidence compared with malignant clavicular tumors [21]. Our study (the average diameter for benign tumors vs. malignant tumors 3.00 ± 1.08 cm vs. 6.23 ± 1.69 cm, *P* < 0.001) confirmed the characteristics described above. Therefore, it is considered that patients can obtain ideal prognosis using intracapsular scraping or tumor resection followed by autologous bone grafting [5, 6]. However, few studies investigated the postoperative function of the shoulder joint. In this study, our patients with clavicular benign tumors underwent intracapsular resection and demonstrated a higher score in the assessment of MSTS, Constant-Murley score, and ASES after 12 months postoperatively. This indicated significant functional improvement of the shoulder joint. Here, our findings provided a new evidence that intracapsular resection is an appropriate treatment option for benign clavicular tumors.

### Surgical method for malignant clavicular tumor

Malignant clavicular tumors present with larger mass, invasive growth, and rapid progress since its first occurrence. Partial or total resection of the clavicle was considered as the optimal therapeutic method. However, whether or not the clavicle should be reconstructed remains controversial and unresolved. Lin et al., Vartanian et al., and Koh et al. [2, 12–14] observed many complications, such as shoulder dropping, postoperative pain, limited motion of the shoulder joint, reduction of muscular strength, neurologic function deficit, and restriction of daily activities, which occurred in patients who underwent claviculectomy without clavicular reconstruction. As a result, the reconstruction of the clavicle after claviculectomy was suggested in order to reduce the incidence of complications. In contrast, some researchers [3, 9, 23, 24] demonstrated that clavicular reconstruction is unnecessary because there are fewer postoperative complications and more satisfaction with surgical outcomes observed in short- to medium-term follow-up care. Kapoor et al. [3] conducted a 6-month to 10-year follow-up program with 12 clavicular tumors patients. In their study, among the 12 patients with clavicular tumors, 7 did not undergo reconstruction after claviculectomy (4 partial and 3 total resection) and they presented with approximately 70.5% normal function in the postoperative functional assessment using the MSTS score. In the review of Ke et al. [1], 206 East Asian cases of primary clavicle tumors and tumorous lesions were analyzed. The authors suggest that to restore the symmetry of the lower neck and upper chest, a simple way to rebuild the clavicle is recommended for adolescents with less growth potential. Additionally, the authors have the perspective that complex clavicular reconstruction is not recommended, especially for pedicled fibular flap grafts, since most upper limb function is preserved after subtotal claviculectomy, total claviculectomy, or curettage. According to the results of the functional assessments, our study indicated that no significant improvement of shoulder joint function was observed compared with the non-reconstruction and reconstruction groups. Furthermore, we found that among the 5 patients who underwent clavicular reconstruction after removing the malignant tumors, 2 were re-operated to fix the loosening screws and plate displacement. In addition, the patients took a longer time to recover their upper limb function [25]. As we know, the plate-cement complex is allogeneic, the cortex of manubrium sterni is thin, and the structure of the acromion is complex. We speculate that these are the risk factors causing damage to the screws and plates after the reconstruction. Our study also showed that there were 2 cases of recurrence and 2 cases of metastasis in the reconstruction group among all cases of recurrence/metastasis of clavicular malignant tumors (2 recurrences and 3 metastases). Thus, we believe that reconstruction may be a factor in the recurrence/metastasis in the clavicular tumors. The range of tumor resection is always insufficient when ensuring adequate space for plate fixation during surgery, and reconstruction requires longer surgery time which may increase the possibility of tumor cells contaminating surrounding tissues and causing postoperative recurrence. However, due to the small sample size of our study, whether or not the recurrence/metastasis of a clavicular tumor is related to reconstruction or histologic type still needs to be confirmed through more studies. We suggest that reconstruction of the clavicle after resection of a malignant tumor is not recommended. However, due to the small sample size of our study and the limited number of available clinical studies, further investigations are required to confirm whether the long-term outcome of clavicular reconstruction is more advantageous than no reconstruction.

## Conclusion

The clavicular tumor is a rare bone disease. Surgery is considered as an optimal treatment to significantly restore shoulder joint function after a short- to medium-term follow-up period. In patients with benign clavicular tumors, simple intracapsular resection can obtain a satisfactory prognosis. We suggest that reconstruction of the clavicle after resection of a malignant tumor is not recommended.

## Data Availability

All data used in the study are available from the corresponding authors.

## Abbreviations

ASES: American Shoulder and Elbow Surgeons shoulder outcome score
MSTS: Musculoskeletal Tumor Society score
